# National Poison Center Calls Before vs After Availability of High-Dose Acetaminophen (Paracetamol) Tablets in Switzerland

**DOI:** 10.1001/jamanetworkopen.2020.22897

**Published:** 2020-10-28

**Authors:** Adrian Martinez-De la Torre, Stefan Weiler, Dominic Stefan Bräm, Samuel S. Allemann, Hugo Kupferschmidt, Andrea M. Burden

**Affiliations:** 1 Institute of Pharmaceutical Sciences, Department of Chemistry and Applied Biosciences, Swiss Federal Institute of Technology Zurich, Zurich, Switzerland; 2 National Poisons Information Centre, Tox Info Suisse, Associated Institute of the University of Zurich, Zurich, Switzerland; 3 Swiss Association of Pharmacists, Bern-Liebefeld, Switzerland

## Abstract

**Importance:**

Acetaminophen (paracetamol) is among the most widely used pain medications worldwide; while safe within the therapeutic range, intake exceeding 4000 mg/d can lead to hepatotoxicity. Prior evidence suggests that limiting the availability of large quantities of acetaminophen is associated with decreased acetaminophen-related poisonings and mortality; in Switzerland, 500-mg tablets are available over-the-counter (OTC) and, as of October 2003, 1000-mg tablets are available with prescription.

**Objective:**

To evaluate the association of adding 1000-mg acetaminophen tablets to the Swiss market with utilization and poisonings.

**Design, Setting, and Participants:**

This cross-sectional study used a quasi-experimental interrupted time series analysis to evaluate 15 790 acetaminophen poison records from January 1, 2000, to December 31, 2018. All calls for acetaminophen-related poisonings identified from the National Swiss Poisons Centre and all sales for oral acetaminophen tablets (prescription and OTC) dispensed between January 2000 and December 2018 were included.

**Exposure:**

October 3, 2003 (Q4 2003), was defined as the intervention date, corresponding to the date of market entry for 1000-mg acetaminophen tablets in Switzerland.

**Main Outcomes and Measures:**

The primary outcome was the number of quarterly acetaminophen-related poison calls to the National Poison Centre. Additional outcomes included quarterly sales for acetaminophen and change in poisoning circumstances, stratified by preintervention and postintervention periods and by formulation (ie, 500-mg and 1000-mg tablets).

**Results:**

Between 2000 and 2018, 15 790 acetaminophen-related poisoning calls were identified, of which 10 628 (67.3%) were regarding women, and the mean (SD) age of patients was 25.2 (18.2) years. The interrupted time series analysis identified a significant increase in the slope for the number of reported poisonings following the intervention point, particularly for accidental circumstances (*z *score, −3.62; *P* < .001). In the preintervention period, 120 of 961 poisonings (15.3%) involved a dose greater than 10 000 mg, while for the postintervention period, 1140 of 5696 (30.6%) had a dose larger than 10 000 mg (*P* < .001). There was a rapid uptake in 1000-mg acetaminophen sales, while sales of the 500-mg tablet decreased slightly. Since 2012, a mean (SD) of 20.7 million (1.4 million) 1000-mg tablets were dispensed quarterly compared with 2.7 million (0.5 million) 500-mg tablets.

**Conclusions and Relevance:**

This study found a significant increase in acetaminophen dispensing and acetaminophen-related poisonings in Switzerland following the approval of 1000-mg tablets in 2003. The availability of 1000-mg acetaminophen should be re-evaluated to minimize the potential for accidental poisonings.

## Key Points

**Question:**

Was the introduction of the 1000-mg formulation of oral acetaminophen tablets associated with an increased number of acetaminophen-related calls to the National Poison Centre in Switzerland?

**Findings:**

This cross-sectional study using a quasi-experimental interrupted time series analysis of 15 790 acetaminophen-related poisoning calls identified a significant increase in the number of calls following the date 1000-mg tablets were authorized, particularly for calls regarding accidental poisonings. The reported poisonings in the postintervention period were more likely to include doses exceeding 10 000 mg, indicating potential for severe hepatoxicity.

**Meaning:**

The results of this study support the need for public health measures to restrict the availability of the 1000-mg acetaminophen tablet to minimize the potential for accidental acetaminophen-related harm.

## Teaser

This cross-sectional study evaluates the association of adding 1000-mg acetaminophen tablets to the Swiss market with utilization and poisonings.

## Introduction

Acetaminophen (paracetamol) is an antipyretic and analgesic drug with a weak anti-inflammatory action. Since its clinical introduction in 1955, acetaminophen has become the most widely used analgesic-antipyretic^1^ and accounts for nearly half of all pain medication prescriptions in Switzerland.^2^ Worldwide, acetaminophen is widely available via prescription or over the counter (OTC), with the most common formulations being 500-mg and 1000-mg oral tablets. The therapeutic dose for those aged 12 years and older is 1000 mg every 4 to 6 hours, with a maximum daily dose of 4000 mg. Among children younger than 12 years, weight-based dosing is recommended (ie, 10-15 mg/kg every 4 to 6 hours), with a maximum daily dose of 75 mg/kg.^3^ When used within this therapeutic dosage, acetaminophen is considered among the safest pain medications available.^4^ As such, it is indicated for the management of mild to moderate pain and/or fever.^4^ However, daily doses exceeding the therapeutic maximum can cause hepatoxicity, and severe toxic effects can occur with doses exceeding 10 000 mg in adults or 150 to 200 mg/kg in children.

In many Western countries, acetaminophen poisoning is the leading cause of liver failure,^5^ and researchers have identified that prolonged exposure to high daily doses can increase the risk of liver failure and death.^6^ Thus, in light of the limited evidence of a clinical benefit for musculoskeletal conditions, headache and migraine, or dental pain,^7,8,9,10^ concerns regarding the benefit-risk profile of acetaminophen have been raised.^11^ In August 2020, the National Institute for Health and Care Excellence (NICE) released draft guidance on chronic pain management, in which acetaminophen (paracetamol) is no longer recommended because of its unfavorable benefit-risk profile.^12^

Access to high doses of acetaminophen may increase the risk of unintentional poisonings owing to a combination of underestimating the risks and lack of clinical effect in pain management, leading to up-dosing.^13^ Several studies have linked OTC acetaminophen availability to higher acetaminophen-related inquiries to poison information centers and acetaminophen-related mortality.^14,15^ As a result, many European countries—including the United Kingdom, Denmark, and Sweden—have implemented pack size restrictions for OTC sales.^1,16^ However, changes in legislations for OTC restrictions have so far only identified modest reductions in mortality and poisonings in some regions, and the association with liver transplantation remains limited.^17,18^

Nevertheless, studies indicate that the use of acetaminophen is increasing.^1,19,20^ This is despite limited evidence of the benefit of acetaminophen in pain management^8^ and accumulating safety concerns with high doses, including hepatoxicity, major cardiovascular events, bleeding, and upper gastrointestinal complications.^6,8^ In Switzerland, acetaminophen is available as 500-mg tablets OTC and with prescription, and as of October 2003, 1000-mg tablets are available with a prescription. Therefore, we sought to characterize and examine the number of acetaminophen-related poisoning reports in Switzerland before and after the introduction of the 1000-mg tablets using quasi-experimental interrupted time series (ITS) analysis. Additionally, we sought to investigate the characteristics of reported acetaminophen poisonings and the total acetaminophen sales in Switzerland, stratified by formulation.

## Methods

### Data Sources

In Switzerland, nationwide (and complementary) consulting to the general public and doctors on poisonings resulting from both drugs and substances is provided by the Swiss National Poison Centre (Tox Info Suisse).^21^ Calls regarding suspected poisonings are received by medical professionals specially trained in clinical toxicology. In 2018, Tox Info Suisse received more than 41 000 calls from the general public (27 287 [66.3%]), medical professionals (10 584 [25.8%]), and other sources (3285 [7.9%]).^21^ Among all calls, approximately one-third were for reported poisonings with pharmaceutical products. For all calls related to pharmaceutical drugs, basic demographic (age, sex, region) and clinical (drug type, route of administration, other drugs ingested, and circumstance of exposure) data are systematically collected and standardized by a clinical toxicologist. Additionally, among cases sent to hospitals or medical practices for clinical care, the treating physician may provide a follow-up report. These additional files contain further information detailing the ingested dose of all substances, intentionality, clinical findings, severity, causality assessment, treatment course (eg, use of antidotes), and clinical outcomes. Standard definitions for outcomes are used, and personnel entering the data are specially trained. Each case is reviewed by a senior clinical toxicologist to ensure completeness and correctness of the entered data. Exceptional cases are discussed by an internal expert panel with clinical toxicologists and poison information specialists present, and discrepancies are resolved by consensus before being entered into the database.

The National Pharmacists Association collects information on all drugs sold at community pharmacies (OTC and prescription). This includes the formulation, drug name, and total number of units (pills) dispensed. The use of Tox Info Suisse patient data was approved by the Swiss Cantonal Ethics Commission. A waiver of informed consent was provided related to the practicability of clinical toxicological studies and the use of deidentified data. This study followed the Strengthening the Reporting of Observational Studies in Epidemiology (STROBE) reporting guideline.

### Study Design

We conducted a quasi-experimental ITS analysis using population-based cross-sectional data from Tox Info Suisse. ITS analysis is considered among the strongest quasi-experimental designs to examine whether an intervention (eg, a policy change) is associated with changes in the data pattern. The ITS analysis assesses the association of an intervention with an outcome of interest by using the preintervention period data to estimate the postintervention period trend.^22^ Cross-sectional data on the number of unique calls (poisonings) for acetaminophen poisonings were extracted from the Tox Info Suisse between January 1, 2000, and December 31, 2018. We excluded all calls originating from outside Switzerland or with unknown origin and calls for non–oral tablet formulations. Additionally, calls with follow-up information were identified and included in a subgroup analyses. As a comparator, we extracted all unique calls to Tox Info Suisse during the same period for ibuprofen. The use of a comparator group that was not exposed to the intervention serves as a counterfactual, whereby the population is as similar as possible but did not experience the intervention of interest. This helps minimize the potential for confounding factors influencing changes in the outcome of interest.^22^ Ibuprofen was selected as a suitable control because it has similar indications and is available both OTC and with prescription but did not undergo any policy change during the study period.

Additionally, cross-sectional data on the national sales for all oral acetaminophen tablets were provided by year and month between January 1, 2000, and December 31, 2018, stratified by dose strength (ie, 500 mg vs 1000 mg). We excluded all sales for nontablet formulations.

### Statistical Analysis

The primary outcome of interest was number of calls for reported acetaminophen poisonings made to the National Poison Centre. To evaluate the association of introducing the 1000-mg oral acetaminophen tablets in Switzerland with the outcome of interest, we used an ITS analysis. In this analysis, segmented linear regression using ordinary least squares estimated the number of quarterly poisonings that may have occurred if 1000-mg acetaminophen had not been introduced to the market. We defined our intervention point as quarter 4 of 2003 (Q4 2003), which corresponds to the date 1000-mg oral tablets entered the market (ie, October 3, 2003). The preintervention period included 15 points before the introduction of 1000-mg tablets (Q1 2000 to Q3 2003), while the postintervention period included 61 points after the introduction of 1000-mg tablets (Q4 2003 to Q4 2018), thereby providing sufficient power to estimate the regression coefficients.^23^ Additionally, we conducted a change-point analysis to identify the point when a significant change, if any, occurred.^24^ In secondary analyses, we replicated the primary ITS analyses (overall and stratified by poisoning circumstance), using only calls in which only acetaminophen was reported (monointoxications) and using a quadratic fit. Additionally, the ITS model with the intervention date of Q4 2003 was applied to calls for ibuprofen poisonings. Details of the ITS model are provided in the eAppendix in the Supplement.

From the National Poison Centre data, we summarized patient demographic and poison characteristics as counts and proportions or means and SDs, as appropriate. We report the characteristics overall and stratified by the preintervention and postintervention periods for all calls and among the subset of calls with follow-up information. In those with follow-up data, we report the age and sex distribution, circumstance of poisoning, use of antidote N-acetylcysteine, and severe or fatal outcomes. Additionally, among the monointoxications with follow-up clinical data and a known ingested dose in the postintervention period, we further stratified by formulation strength (ie, 500 mg vs 1000 mg) and total reported ingested dose in mg (ie, ≤4000 mg, >4000 to 9999 mg, and ≥10 000 mg). In a post hoc analysis, we examined the circumstances among children younger than 10 years, stratified by acetaminophen formulation. In a secondary analysis, we report the patient demographic characteristics for all calls with monointoxications with acetaminophen. Missing data were included and reported as individual categories when appropriate. Significant differences between the preintervention and postintervention periods or between the 500-mg and 1000-mg formulations were tested using χ^2^ and *t* tests, as appropriate.

The monthly national pharmacy sales data of acetaminophen tablets were tabulated and plotted by year and calendar quarter (Q1, January to March; Q2, April to June; Q3, July to September; and Q4, October to December) and stratified by tablet strength (ie, 500 mg vs 1000 mg). Similarly, all calls reported to the National Poison Centre data for acetaminophen and ibuprofen were tabulated and plotted by year and quarter.

All analyses were performed in R version 3.5.1 (R Project for Statistical Computing). Statistical significance was set at *P* < .05, and all tests were 2-tailed. When designing the studies, we followed recommendations by Jandoc et al^22^ on ITS analysis design.

## Results

**Figure 1.**
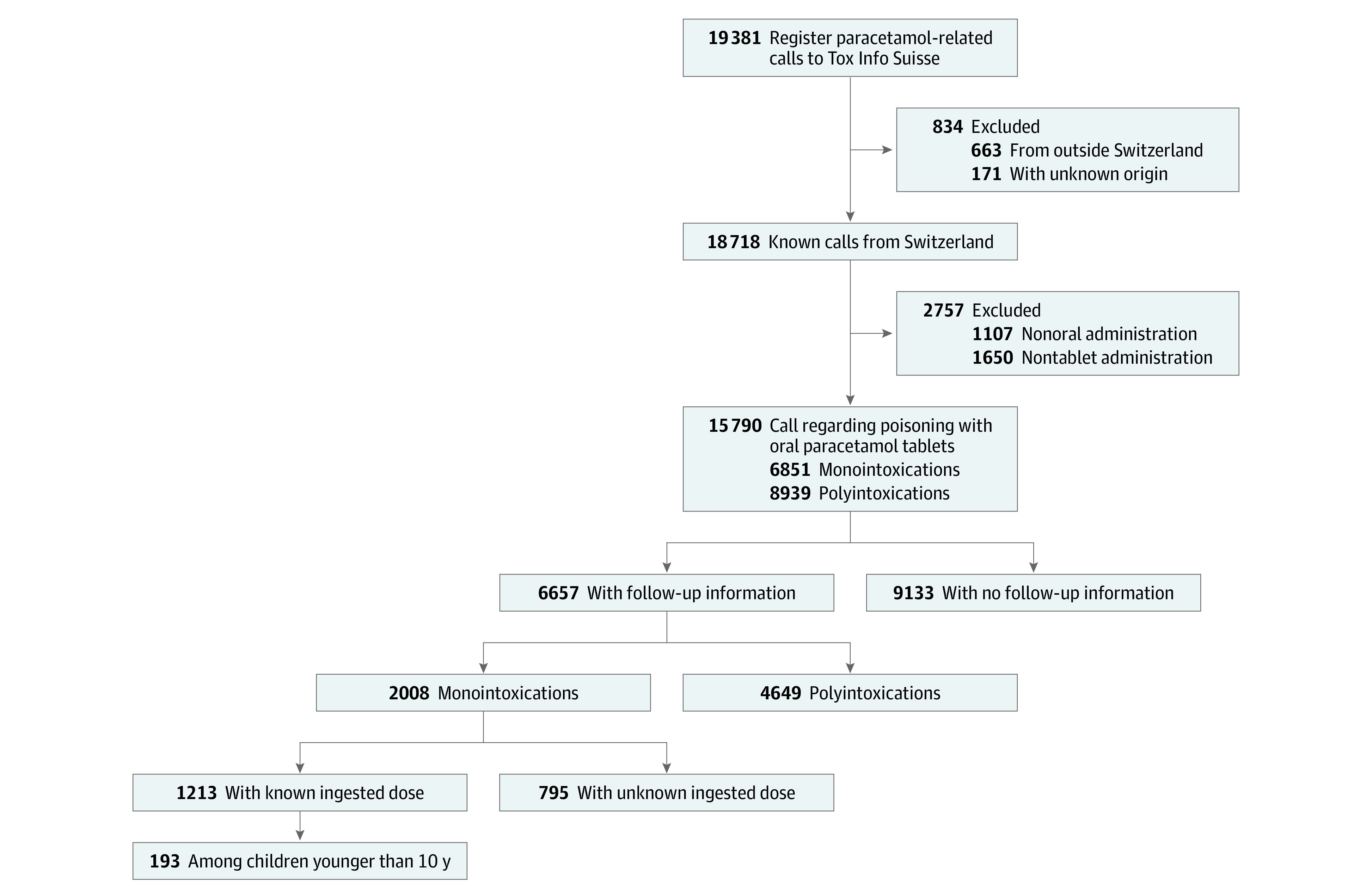
Flowchart of the Inclusion Criteria for the Tox Info Suisse Poison Data

Following exclusions, a total of 15 790 eligible acetaminophen poisonings were reported during the study period (Figure 1). Table 1 provides the demographic characteristics of all calls to the National Poison Centre, stratified by preintervention and postintervention periods. Most calls in both periods were regarding women (overall: 10 628 [67.3%]; preintervention: 1395 [67.1%]; postintervention: 9233 [67.3%]) and patients with a mean (SD) age of 25.2 (18.2) years. In both periods, those aged 16 to 24 years made the plurality of calls (preintervention: 562 [27.0%]; postintervention: 3574 [26.1%]). Compared with the preintervention period, the postintervention had a higher proportion of calls for children younger than 6 years (234 [11.3%] vs 1924 [14.0%]; *P* < .001), among those aged 45 to 65 years (115 [5.5%] vs 1404 [10.2%]; *P* < .001), and those older than 65 years (19 [0.9%] vs 471 [3.4%]; *P* < 001). From the preintervention period to the postintervention, the proportion of accidental poisonings and monointoxications increased from 414 (19.9%) to 4185 (30.5%; *P* < .001) and from 618 (29.7%) to 6233 (55.5%; *P* < .001), respectively. The total ingested dose was known in 1480 cases (71.2%) in the preintervention period and 8843 (64.5%) in the postintervention period (*P* < .001). Among those with a known dose, the mean (SD) reported dose increased significantly from 6900 (6600) mg to 9000 (33 500) mg between the preintervention and postintervention periods, respectively (*P* = .02). However, we did not observe a significant increase in the median (interquartile range) ingested dose, which increased from 5000 (2500-10 000) mg in the preintervention period to 6000 (2000-10 000) mg in the postintervention period.

**Table 1.** Demographic Characteristics of 15 790 Calls Reported to the National Poison Centre in Switzerland for Oral Acetaminophen Tablets Between January 2000 and December 2018, Stratified by Intervention Period

Characteristic	No. (%)		
	Overall (N = 15 790)	Preintervention (n = 2079)	Postintervention (n = 13 711)
Sex			
Women	10 628 (67.3)	1395 (67.1)	9233 (67.3)
Unknown	370 (2.3)	154 (7.4)	216 (1.6)
Age, mean (SD), y^a^			
Mean (SD)	25.2 (18.2)	22.7 (14.7)	25.5 (18.6)
<6	2158 (13.7)	234 (11.3)	1924 (14.0)
6-9	128 (0.8)	16 (0.8)	112 (0.8)
10-15	1512 (9.6)	217 (10.4)	1295 (9.4)
16-24	4136 (26.2)	562 (27.0)	3574 (26.1)
25-44	3356 (21.3)	430 (20.7)	2926 (21.3)
45-65	1519 (9.6)	115 (5.5)	1404 (10.2)
>65	490 (3.1)	19 (0.9)	471 (3.4)
Unknown	2491 (15.8)	486 (23.4)	2005 (14.6)
Circumstance of poisoning^a^			
Accidental	4599 (29.1)	414 (19.9)	4185 (30.5)
Intentional	10 844 (68.7)	1611 (77.5)	9233 (67.3)
Adverse event	138 (0.9)	26 (1.3)	112 (0.8)
Unknown	209 (1.3)	28 (1.3)	181 (1.3)
Polyintoxication	8939 (56.6)	1461 (70.3)	7478 (54.5)
Monointoxication	6851 (43.4)	618 (29.7)	6233 (55.5)
Known dose	10 323 (65.4)	1480 (71.2)	8843 (64.5)
Dose, mg			
Mean (SD)^a^	8690 (31 100)	6870 (6580)	9000 (33 490)
Median (IQR)	6000 (2000-10 000)	5000 (2500-10 000)	6000 (2000-10 000)
Dose category, mg^a^^,^^b^			
≤4000	4283 (41.5)	608 (41.1)	3675 (41.6)
>4000 to 9999	3824 (37.0)	663 (44.8)	3161 (35.7)
≥10 000	1673 (16.2)	173 (11.7)	1500 (17.0)
≥25 000	543 (5.3)	36 (2.4)	507 (5.7)

Abbreviation: IQR, interquartile range.

^a^ *P* < .05 between preintervention and postintervention periods.

^b^ Proportions calculated among those with a known dose.

**Figure 2.**
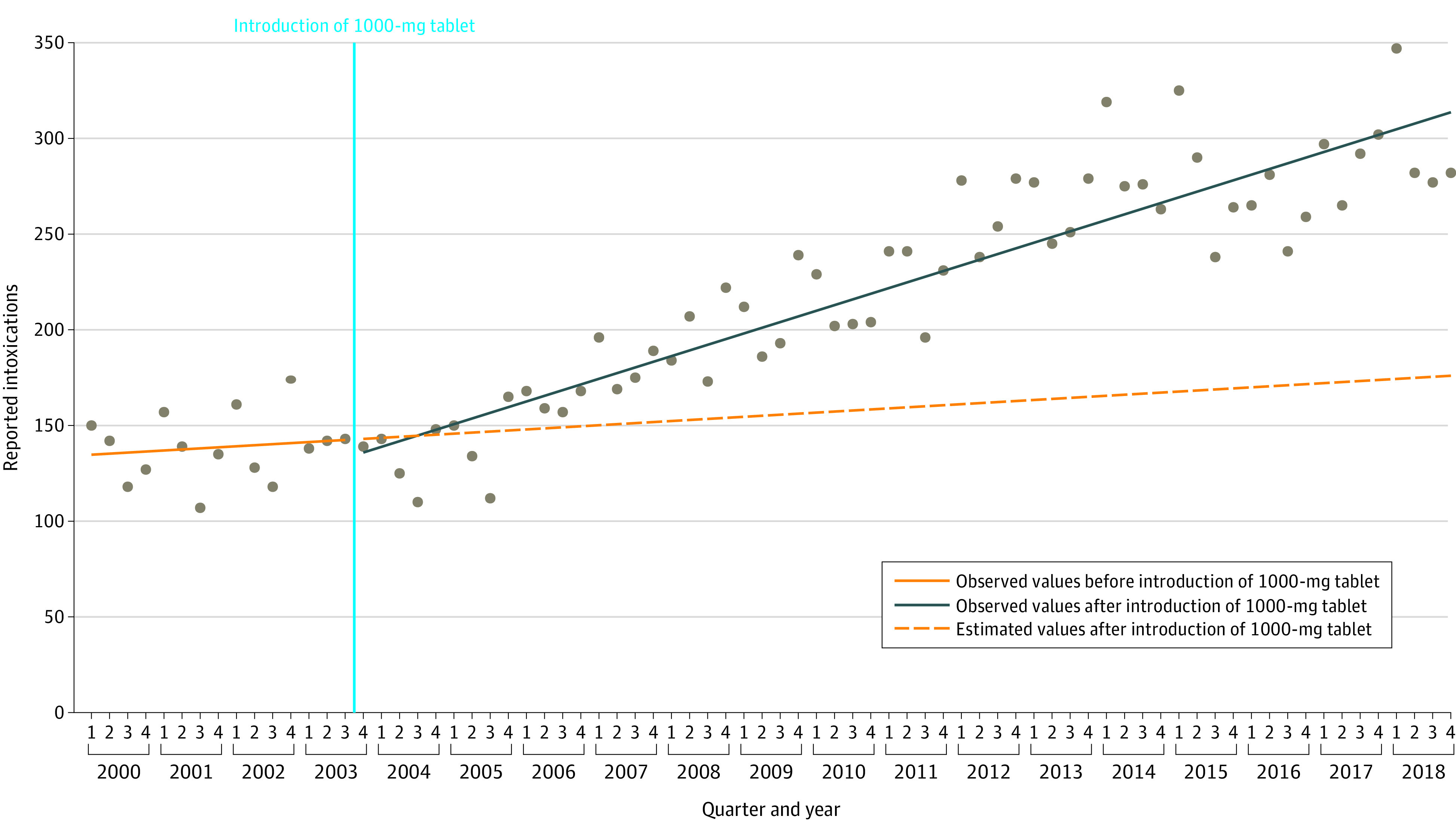
Interrupted Time Series Analysis for All Reported Intoxication Calls for Acetaminophen in Switzerland Between 2000 and 2018 The period to the left of the blue vertical line is the preintervention period, while that to the right is the postintervention period.

**Figure 3.**
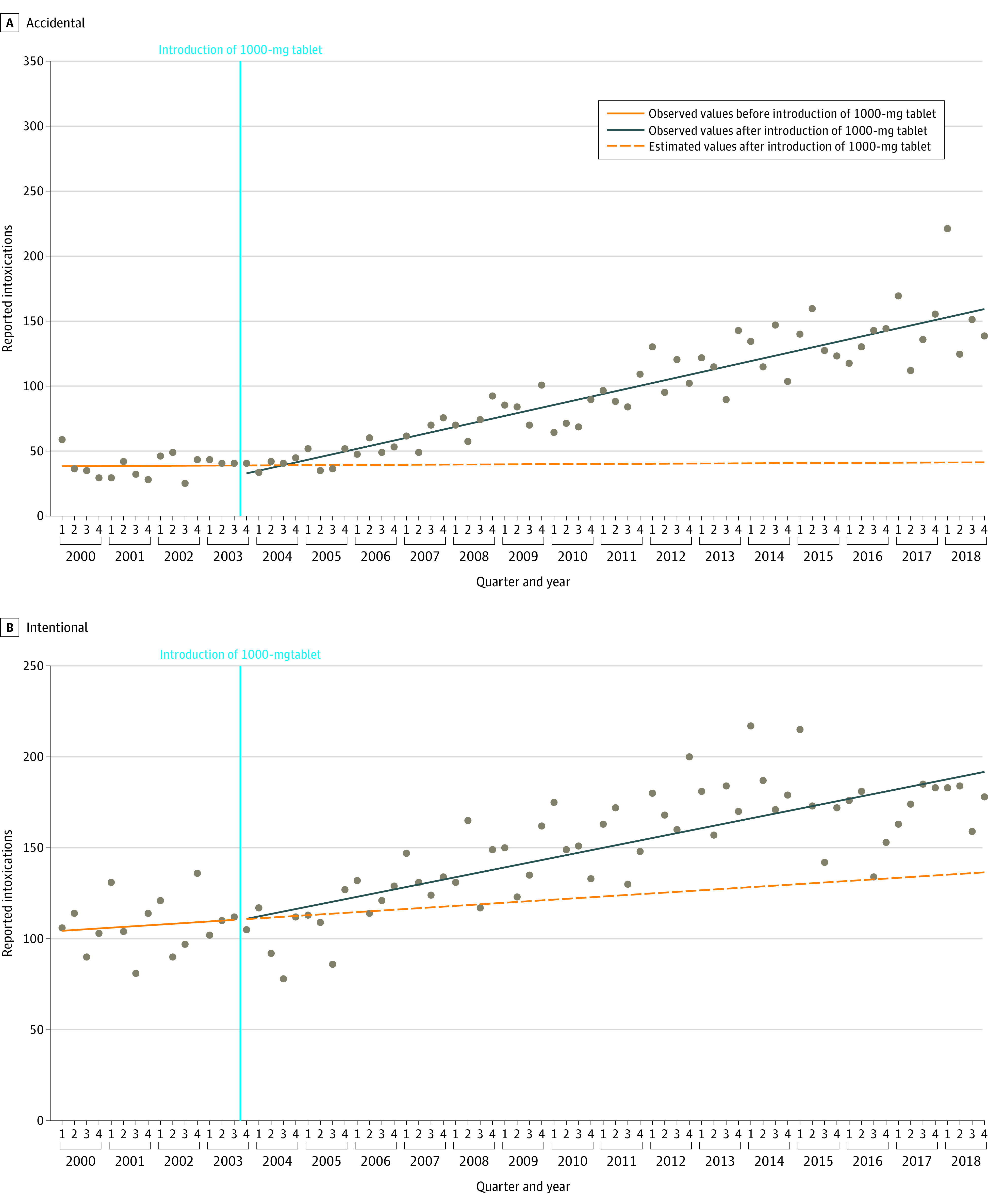
Interrupted Time Series Analysis for Accidental and Intentional Intoxication Calls for Acetaminophen in Switzerland Between 2000 and 2018 In both panels, the period to the left of the blue vertical line is the preintervention period, while that to the right is the postintervention period.

The ITS analysis identified a statistically significant difference between the preintervention and postintervention slopes (*z *score, −3.01; *P* = .002) (Figure 2). While the ITS used Q4 2003 as the intervention point, the change-point analysis identified Q4 2004 as the point at which a statistically significant change in the number of quarterly inquiries occurred. Further details on the regression coefficients can be found in eTable 1 in the Supplement. When stratified by circumstance, we identified a significant difference in the slopes between the preintervention and postintervention period for accidental (*z *score, −3.62; *P* < .001) but not intentional (*z *score, −1.85; *P* = .06) poisonings (Figure 3A and Figure 3B).

A total of 6657 patients (42.1%) had follow-up information available, and the trends observed were similar to those identified among all calls (eTable 2 in the Supplement). We identified an increase in the proportion of calls with reported toxic doses of 10 000 mg or more in preintervention vs postintervention periods (120 [15.3%] vs 1140 [30.6%]; *P* < .001). Additionally, we observed an increase in the proportion of patients receiving the antidote (424 [44.1%] vs 3157 [55.4%]; *P* < .001) and those with a severe outcome (55 [5.7%] vs 406 [7.1%]; *P* < .001).

Table 2 displays the poisoning circumstances, stratified by acetaminophen formulation and the total dose ingested among the 1213 reports with a known dose and monointoxication. Overall, 240 reports (19.8%) were regarding 500-mg tablets, 486 (40.0%) regarding 1000-mg tablets, and 487 (40.2%) regarding an unknown formulation. Among those with exposure between greater than 4000 mg and 9999 mg, 123 of 504 (24.4%) were for 500-mg tablets and 185 (36.7%) for 1000-mg tablets. We observed a significant increase in accidental poisonings for the 1000-mg tablets compared with the 500-mg tablets (81 [43.8%] vs 19 [15.4%]; *P* < .001). However, the antidote was administered more frequently among those who took 500-mg tablets compared with those who took the 1000-mg tablets (65 [52.8%] vs 80 [43.2%]; *P* = .03), and no difference was observed for severe or fatal outcomes. Among those with toxic exposure of 10 000 mg or greater, 91 of 511 (17.8%) were observed among 500-mg tablets and 244 (47.8%) among 1000-mg tablets. The proportion of accidental poisonings was higher among the 1000-mg tablets compared with 500-mg tablets (54 [22.1%] vs 10 [11.0%]; *P* < .001), but no differences were observed for receiving the antidote or clinical outcomes. The post hoc analysis among children younger than 10, 188 of 193 (97.4%) were accidental circumstances (eTable 3 in the Supplement). In 50 cases (25.9%), the child had consumed the 1000-mg tablet, compared with only 31 cases (16.0%) with the 500 mg. However, no significant differences were identified.

**Table 2.** Demographic Characteristics Among 1213 Calls Regarding Monointoxication With Known Cumulative Dose and Follow-up, Reported to the National Poison Centre in Switzerland in the Postintervention Period, by Formulation and Cumulative Dose

Characteristic	No. (%)		
	500-mg tablets (n = 240)	1000-mg tablets (n = 486)	Unknown formulation (n = 487)
**Acetaminophen poisoning with total ingestion of ≤4000 mg (n = 198)**			
No.	26	57	115
Sex			
Women	19 (73.1)	33 (57.9)	62 (53.9)
Unknown	0	1 (1.8)	6 (5.2)
Age, y^a^			
Mean (SD)	10.4 (13.5)	21.05 (23.9)	17.84 (21.1)
0-9	16 (61.5)	24 (42.1)	57 (49.6)
10-24	7 (26.9)	17 (29.8)	25 (21.8)
25-65	3 (3.8)	12 (21)	27 (23.5)
≥65	0	4 (7.0)	5 (4.3)
Unknown	0	0	1 (0.9)
Circumstance			
Accidental	17 (65.4)	39 (68.4)	68 (59.1)
Intentional	9 (34.6)	17 (29.8)	45 (39.1)
Unknown	0	1 (1.8)	2 (1.7)
N-acetylcysteine antidote			
Yes	8 (30.8)	17 (29.8)	44 (38.3)
No	18 (69.2)	40 (70.2)	68 (59.1)
Unknown	0	0	3 (2.6)
Severity			
Severe symptoms	0	1 (1.8)	4 (3.5)
Died	0	0	2 (1.7)
**Acetaminophen poisoning with total ingestion of >4000 mg-9999 mg (n = 504)**			
No.	123	185	196
Sex			
Women^a^	17 (13.8)	53 (28.6)	53 (27.0)
Unknown	1 (0.8)	0	2 (1.0)
Age, y^a^			
Mean (SD)	25.09 (14.8)	30.64 (18.3)	26.39 (14.6)
0-9	0	2 (1.1)	2 (1.0)
10-24	77 (62.6)	94 (50.8)	110 (56.2)
25-65	40 (32.5)	79 (42.7)	74 (37.7)
>65	5 (4.1)	10 (5.4)	3 (1.5)
Unknown	1 (0.8)	0	7 (3.6)
Circumstance^a^			
Accidental	19 (15.4)	81 (43.8)	40 (20.4)
Intentional	101 (82.1)	102 (55.1)	155 (79.1)
Unknown	3 (2.4)	2 (1.1)	1 (0.5)
N-acetylcysteine antidote			
Yes	65 (52.8)	80 (43.2)	112 (57.1)
No	56 (45.5)	101 (54.6)	81 (41.3)
Unknown	2 (1.6)	4 (2.2)	3 (1.5)
Severity			
Severe symptoms	2 (1.6)	2 (1.1)	1 (0.5)
Died	1 (0.8)	0	0
**Acetaminophen poisoning with total ingestion of ≥10 000 mg (n = 511)**			
No.	91	244	176
Sex			
Women	72 (79.1)	178 (73.0)	142 (80.7)
Unknown	0	0	0
Age, y			
Mean (SD)	26.45 (13.8)	29.18 (17.6)	29.42 (16.1)
0-9	0	0	0
10-24	57 (62.6)	132 (54.1)	82 (46.6)
25-65	32 (35.2)	100 (41.0)	87 (49.4)
>65	2 (2.2)	12 (4.9)	7 (4.0)
Circumstance^a^			
Accidental	10 (11.0)	54 (22.1)	12 (6.8)
Intentional	81 (89.0)	189 (77.5)	162 (92.0)
Unknown	0	1 (0.4)	2 (1.1)
N-acetylcysteine antidote			
Yes	84 (92.3)	229 (93.9)	161 (91.5)
No	7 (7.7)	15 (6.1)	14 (8.0)
Unknown	0	0	1 (0.6)
Severity			
Severe symptoms	6 (6.6)	12 (4.9)	14 (8.0)
Died	1 (1.1)	1 (0.4)	0

^a^ *P* < .05 between the 500-mg and 1000-mg formulations.

In our secondary analyses, including only calls with monointoxications with acetaminophen, we identified similar results for the ITS analysis (eFigure 1A and eFigure 1B in the Supplement) and patient demographic characteristics (eTable 4 in the Supplement) compared with the primary analysis with all calls. Additionally, using a quadratic specification resulted in similar conclusions, overall and stratified by circumstance (eFigure 2, eFigure 3A, and eFigure 3B in the Supplement, respectively). In the comparator analysis with ibuprofen, we identified a significant change in slope following the intervention point (Q4 2003); however, the change-point analysis identified Q4 2007 as the point at which a significant increase in in the mean number of quarterly inquiries occurred (eFigure 4 in the Supplement).

eFigure 5 in the Supplement displays the quarterly acetaminophen sales data, stratified by formulation strength. From the date of introduction there was a rapid uptake of 1000-mg acetaminophen sales, with the total number of 1000-mg tablets sold surpassing the 500-mg sales within 2 years of market entry. The total sales of 1000-mg tablets plateaued in 2012, after which a mean (SD) of 20.8 million (1.4 million) tablets were sold per quarter compared with 2.7 million (0.5 million) 500-mg tablets. Conversely, we observed a 69% decrease in the total number of 500-mg tablets sold, from a total of 7.0 million tablets in Q1 2003 to 2.2 million in Q4 2018.

## Discussion

In this cross-sectional study, we identified a significant increase in reported acetaminophen poisonings in Switzerland. A statistically significant change was observed among accidental but not intentional poisonings. Importantly, the proportion of patients with accidental poisonings increased among those reporting 1000-mg intake compared with the 500-mg formulation in those with ingested doses greater than the therapeutic range of 4000 mg. Moreover, among all calls regarding ingested doses greater than 10 000 mg, most reported intake of the 1000-mg tablets. The national sales data further identified that there was a rapid uptake in 1000-mg tablet sales, which significantly exceed the sales of 500-mg tablets within 2 years of introduction.

In 2008, the US Food and Drug Administration (FDA) recommended limiting the single adult dose of acetaminophen to a maximum of 650 mg (2 × 325 mg acetaminophen tablets) to reduce the incidence of hepatotoxicity.^25^ However, we observed that the number of 1000-mg tablets dispensed grew substantially in Switzerland during this time, despite requiring a prescription from a medical doctor. As the total sales of 1000-mg acetaminophen tablets grew rapidly in the first 9 years of availability, reaching a mean of more than 20 million tablets sold per quarter by 2012, 10 times greater than sales of 500-mg tablets, our results suggest that the 1000-mg tablets did not directly replace the 500-mg formulation. The high use of the 1000-mg tablet may be owing to a number of factors, including an avoidance of more potent pain medications following concerns with cox inhibitors and opioid-related problems, particularly among older patients with chronic pain.^26^ Indeed, it was previously identified that the prescription rates for all pain medications increased following the withdrawal of rofecoxib in Switzerland in 2005.^2^

Nevertheless, the wide utilization of 1000-mg acetaminophen in Switzerland is concerning considering the ongoing debate regarding the evidence of effectiveness.^7,8,27,28^ A 2011 meta-analysis^29^ found that acetaminophen has a marginal benefit compared with placebo in acute pain, and NICE recently released guidance removing acetaminophen from the list of recommended treatments for chronic pain management because of a lack of effectiveness and increased risk of harm.^12^ In addition, it has been shown that acetaminophen users were more likely to remedicate early, thereby suggesting a lack of effectiveness and a potential for unintentional poisoning.^30^

The limited effectiveness of acetaminophen for pain, particularly chronic pain, is important to consider in light of the potential for toxic effects with large quantities reaching supratherapeutic dosages greater than the maximum daily dose of 4000 mg. While this maximum daily dose threshold is debated, with some experts recommending it be reduced to 3250 mg,^25^ the evidence of the potential for liver damage above 10 000 mg is clear. In our analysis, among calls with acetaminophen monointoxications and information on the ingested dose, we observed that the 1000-mg tablets were overrepresented among those with exposure exceeding 10 000 mg. While we did not observe differences in deaths or severe outcomes, we note that more than 90% of patients with exposure greater than 10 000 mg received the antidote, thereby minimizing the likelihood of severe negative outcomes.

The ITS analysis revealed a significant increase in the reporting of acetaminophen-related calls to the National Poison Centre within 1 year of the 1000-mg tablet being added to the market. While the total number of calls was small in comparison with the total tablets sold, particularly following 2012, these results suggest that adding a more potent formulation of acetaminophen was associated with increased harm. This result is in line with previous studies showing that reducing the available pack sizes of acetaminophen may lead to sustained reductions in the total number of poisonings.^16,17,31^ However, while Hawton and colleagues^17^ identified that pack size restriction legislation in the United Kingdom was associated with a decrease in acetaminophen-related deaths, they did not observe a reduction in liver transplantation registration or transplantation. Moreover, while legislation limiting prescription medications has led to changes in utilization patterns, a study by Bateman et al^32^ concluded that legislations limiting OTC drugs were unlikely to be successful. Thus, given that the 1000-mg acetaminophen tablets are currently only available with a medical prescription, we could expect a greater impact of regulations limiting (or withdrawing) the 1000-mg tablet than in studies analyzing the impact of limiting pack sizes of the 500-mg OTC formulations.

While intentional poisonings represented most cases in our data set and a slight (nonsignificant) increase in calls was observed following the introduction of 1000-mg tablets, the largest difference in the preintervention vs postintervention periods was among accidental poisonings. Importantly, we identified a significant increase in accidental poisoning following the introduction of the 1000-mg tablets in Switzerland, and the proportion of accidental poisonings was larger in patients reporting ingestion of 1000-mg tablets than those reporting ingestion of 500-mg tablets. In particular, we observed that among poisonings with exposures greater than the adult therapeutic threshold (ie, >4000 mg), the proportion of accidental poisonings was doubled among those taking 1000-mg tablets. Thus, while we acknowledge the importance of intentional poisonings, we are alarmed by the significant increase in accidental cases in the postintervention period and among 1000-mg tablets because these are potentially preventable cases. These results are similar to a previous study from Scotland, in which unintentional overdoses vs intentional overdoses represented fewer hospital admissions but greater organ dysfunction and higher mortality (38% vs 26%, respectively).^11^

In the case of 1000-mg tablets, accidental poisonings with acetaminophen could be because patients exceed the maximum daily dose because of insufficient pain control or because they mistook the 1000-mg tablets for the 500-mg formulation, thereby accidentally doubling their expected intake. These mistakes can be mitigated through medical consultation at the time of prescribing and dispensing. Considering that the 1000-mg tablets require a medical prescription and both the 500-mg and 1000-mg tablets are only available within pharmacies, there is ample opportunity in Switzerland to improve the public health measures to minimize the risk of accidental acetaminophen poisonings in adults. However, we also observed an elevated proportion of calls related to poisonings in children younger than 10 years in the postintervention period. Notably, in 50 of 193 cases (25.9%) with clinical follow-up, the child had consumed the 1000-mg tablet compared with only 31 cases (16.0%) with the 500-mg tablet. While we are limited by missing information on the formulation, we are cognizant that the availability of 1000-mg tablets in households may increase the risk of accidental poisonings in young children, for whom a single tablet may exceed the daily maximum.

The risk of unintentional poisonings and subsequent hepatoxicity and increased health care costs with acetaminophen may also be elevated among the frail, older adults, and those with comorbidities (eg, chronic pain, malnutrition, alcohol use disorder, chronic liver disease, or an accompanying medical illness resulting in low reserves of hepatic glutathione). In older patients with a high comorbidity burden, 1000-mg acetaminophen may be prescribed for the convenience of fewer tablets and under the assumption of being the least harmful. However, considering the lack of effectiveness, patients may remedicate early, thereby increasing the risk of unintentional overdose. In our study, we observed that the proportion of older patients with poisonings, particularly poisonings with exposures greater than 4000 mg, was elevated in the postintervention period and among those with 1000-mg tablets, suggesting these patients may be at an elevated risk.

The potential for harm with the 1000-mg tablets is particularly important when considering the observed high-prescribing rate to community-dwelling individuals, among whom unintentional poisonings may be more likely to occur than among patients living in institutions. Given the lack of effectiveness evidence and the potential for harm, particularly at high doses, strategies to reduce availability in the community are needed to minimize harm, particularly to avoid accidental cases. The recent NICE guidelines removing paracetamol from chronic pain management is a first step. However, imposing strict nationwide prescribing restrictions, limiting the use of the 1000-mg tablet to those patients monitored by medical professionals (eg, in long-term-care and nursing homes, during in-patient hospitalization, or those with prepared adherence packages at the pharmacy) is recommended.

### Limitations

While our study was population-based, we interpret our results in light of the inherent limitations. We have a relatively small sample size and note that we might have underestimated the total amount of acetaminophen-induced harm, given that only calls made to the National Poison Centre in Switzerland were included. However, we note that calls to a national poison center, particularly those with follow-up clinical information, can provide useful and needed information on the population poisoning trends, particular because there is known underreporting of drug overdoses (poisonings) to the Swiss Agency for Therapeutic Products (Swissmedic).^21^ Another limitation was that our primary end point (poisoning) was identified as calls to the Tox Info Suisse for a suspected acetaminophen poisoning rather than a laboratory-confirmed diagnosis. While we did not have data on laboratory-confirmed acetaminophen levels or hepatoxicity diagnoses, we were able to identify key clinical outcomes, including supratherapeutic and toxic doses (ie, >4000 mg or ≥10 000 mg), use of antidotes, and the development of severe symptoms or fatal cases. In Switzerland, antidotal therapy is performed parenterally for 20 hours, requiring hospitalization. Considering the severity of outcomes (liver failure, transplantation, or fatality) after toxic doses mainly depends on the timely administration of the antidotal therapy, our measure of severity may not be a direct marker of outcomes associated with high intake because individuals who received the antidote may not develop severe or fatal symptoms. Moreover, long-term effects associated with chronic or repetitive use of high-doses are not covered in our database but should be studied in the future to assess the effects in relation to diagnosis, the correct and timely treatment course, and outcomes.

We note that the results of our study are primarily relevant to the Swiss population. However, while we use data from Switzerland, our results are applicable to other countries where 1000-mg tablets are available, including Spain and France. Indeed, we note that France has recently taken steps to limit the availability of acetaminophen to the public by placing packages (including of 1000-mg tablets) behind the counter.^33^ However, our data would suggest these measures may not be sufficient. Thus, more substantial restrictions on use are likely required. Additionally, it is possible that an increase in calls may be expected with the availability of a new medication, thereby leading to a potential reporting bias. We were also limited by missing data, particularly when evaluating the formulation and dose. In particular, it is possible that the trends observed when comparing the 500-mg tablets with the 1000-mg tablets could differ if there were no missing data; however, we have no evidence to suggest a systematic difference in the completeness of reporting between the 1000-mg and the 500-mg cases. Therefore, we would expect this to be missing at random. Nevertheless, it precludes us from drawing conclusions on the missing data. Furthermore, inaccuracies in the total ingested dose are possible. To minimize errors, we only calculated this among those with monointoxications so that we could be certain of the dose-to-drug relationship in the data. Finally, our sales data were derived from community pharmacy sales in Switzerland. As such, we were unable to identify patient characteristics or include acetaminophen sold through in-office physician dispensing or provided in hospitals.

## Conclusions

In this cross-sectional study, the number of acetaminophen-related poisonings increased following the introduction of the 1000-mg oral acetaminophen tablets. This increase was significant for the number of accidental poisonings, suggesting patients unintentionally exceeded the maximum daily dose. Importantly, our analysis revealed that the proportion of potentially severe hepatotoxic doses (ie, ≥10 000 mg) was highest among those using the 1000-mg tablets compared with those using the 500-mg tablets, particularly among accidental cases. While the 1000-mg tablets may offer the convenience of fewer tablets in a single ingestion for some patients (eg, nursing homes or high polypharmacy), the question of whether convenience should outweigh the potential for harm at the community level should be re-evaluated. Worldwide, 500-mg oral acetaminophen tablets are available and can be dose adjusted to reach individual administrations of 1000 mg when medically required. Thus, with the accumulating evidence of the potential for harm with high doses of acetaminophen, it is pivotal that public health measures aimed at restricting the availability of 1000-mg acetaminophen tablets be considered.

## Notes

Supplement. **eAppendix. **Detailed Methodology on Interrupted Time Series Model **eFigure 1. **Interrupted Time Series Analysis for Reported Accidental and Intentional Monointoxication Calls for Acetaminophen in Switzerland Between 2000 and 2018 **eFigure 2. **Interrupted Time Series Analysis With a Quadratic Specification for All Reported Intoxication Calls for Acetaminophen in Switzerland Between 2000 and 2018 **eFigure 3. **Interrupted Time Series Analysis With a Quadratic Specification for Reported Accidental and Intentional Monointoxication Calls for Acetaminophen in Switzerland Between 2000 and 2018 **eFigure 4. **Interrupted Time Series Analysis for All Reported Intoxication Calls for Ibuprofen in Switzerland Between 2000 and 2018 **eFigure 5. **Total Acetaminophen Tablet Sales in Switzerland, Stratified by Formulation and Year **eTable 1. **Beta Coefficients for Interrupted Time Series Analysis, Stratified by Circumstance and Preintervention and Postintervention **eTable 2. **Demographic Characteristics of 6657 Follow-up Calls Reported to the National Poison Centre in Switzerland for Oral Acetaminophen Tablets Between January 2000 and December 2018, Stratified by Intervention Period **eTable 3. **Demographic Characteristics of 193 Children Aged 0 to 9 Years Among Follow-up Monointoxication Calls Reported to the Nation Poison Centre in Switzerland in the Postintervention Period, Stratified by Acetaminophen Formulation **eTable 4. **Demographic Characteristics of 6851 Monointoxication Calls Reported to the National Poison Centre in Switzerland for Oral Acetaminophen Tablets Between January 2000 and December 2018, Stratified by Intervention Period
