# Supplementary material for: National Poison Center Calls Before vs After Availability of High-Dose Acetaminophen (Paracetamol) Tablets in Switzerland
Source: JAMA Netw Open. 2020 Oct 28;3(10):e2022897. doi: 10.1001/jamanetworkopen.2020.22897 (PMC7593813; doi:10.1001/jamanetworkopen.2020.22897)
Supplement: Supplement. — eAppendix. Detailed Methodology on Interrupted Time Series Model eFigure 1. Interrupted Time Series Analysis for Reported Accidental and Intentional Monointoxication Calls for Acetaminophen in Switzerland Between 2000 and 2018 eFigure 2. Interrupted Time Series Analysis With a Quadratic Specification for All Reported Intoxication Calls for Acetaminophen in Switzerland Between 2000 and 2018 eFigure 3. Interrupted Time Series Analysis With a Quadratic Specification for Reported Accidental and Intentional Monointoxication Calls for Acetaminophen in Switzerland Between 2000 and 2018 eFigure 4. Interrupted Time Series Analysis for All Reported Intoxication Calls for Ibuprofen in Switzerland Between 2000 and 2018 eFigure 5. Total Acetaminophen Tablet Sales in Switzerland, Stratified by Formulation and Year eTable 1. Beta Coefficients for Interrupted Time Series Analysis, Stratified by Circumstance and Preintervention and Postintervention eTable 2. Demographic Characteristics of 6657 Follow-up Calls Reported to the National Poison Centre in Switzerland for Oral Acetaminophen Tablets Between January 2000 and December 2018, Stratified by Intervention Period eTable 3. Demographic Characteristics of 193 Children Aged 0 to 9 Years Among Follow-up Monointoxication Calls Reported to the Nation Poison Centre in Switzerland in the Postintervention Period, Stratified by Acetaminophen Formulation eTable 4. Demographic Characteristics of 6851 Monointoxication Calls Reported to the National Poison Centre in Switzerland for Oral Acetaminophen Tablets Between January 2000 and December 2018, Stratified by Intervention Period [file jamanetwopen-e2022897-s001.pdf]

Supplemental Online Content

Martinez-De la Torre A, Weiler S, Bräm DS, Allemann SS, Kupferschmidt H, Burden AM. National poison center calls before vs after availability of high-dose acetaminophen (paracetamol) tablets in Switzerland. *JAMA Netw Open*. 2020;3(10):e2022897. doi:10.1001/jamanetworkopen.2020.22897

eAppendix. Detailed Methodology on Interrupted Time Series Model
eFigure 1. Interrupted Time Series Analysis for Reported Accidental and Intentional Monointoxication Calls for Acetaminophen in Switzerland Between 2000 and 2018
eFigure 2. Interrupted Time Series Analysis With a Quadratic Specification for All Reported Intoxication Calls for Acetaminophen in Switzerland Between 2000 and 2018
eFigure 3. Interrupted Time Series Analysis With a Quadratic Specification for Reported Accidental and Intentional Monointoxication Calls for Acetaminophen in Switzerland Between 2000 and 2018
eFigure 4. Interrupted Time Series Analysis for All Reported Intoxication Calls for Ibuprofen in Switzerland Between 2000 and 2018
eFigure 5. Total Acetaminophen Tablet Sales in Switzerland, Stratified by Formulation and Year
eTable 1. Beta Coefficients for Interrupted Time Series Analysis, Stratified by Circumstance and Preintervention and Postintervention
eTable 2. Demographic Characteristics of 6657 Follow-up Calls Reported to the National Poison Centre in Switzerland for Oral Acetaminophen Tablets Between January 2000 and December 2018, Stratified by Intervention Period
eTable 3. Demographic Characteristics of 193 Children Aged 0 to 9 Years Among Follow-up Monointoxication Calls Reported to the Nation Poison Centre in Switzerland in the Postintervention Period, Stratified by Acetaminophen Formulation
eTable 4. Demographic Characteristics of 6851 Monointoxication Calls Reported to the National Poison Centre in Switzerland for Oral Acetaminophen Tablets Between January 2000 and December 2018, Stratified by Intervention Period

This supplemental material has been provided by the authors to give readers additional information about their work.

eAppendix. Detailed Methodology on Interrupted Time Series Model

The general ITS model specification is presented (equation 1). The outcome measure, Y_t, is the number of calls to Tox Info Suisse; T_t corresponds to the time elapsed since the start of the study; $Post_t$ is a dummy variable which takes value 0 for the preintervention period, and 1 for the post-intervention period; ε_t is the error term.

$$Y_t = \beta_0 + \beta_1 T_t + \beta_2 Post_t + \beta_3 T_t Post_t + \varepsilon_t \quad (1)$$

Equation 1. Interrupted Time Series Model

The autocorrelation function (ACF) and the partial autocorrelation function (PACF) were inspected for all models. Additionally, we calculated the Durbin-Watson (DW) statistic in order to check for autocorrelation of the residuals. The DW test checks the null hypothesis of no autocorrelation of the residuals.[1] We corrected the standard errors of the coefficients by using Heteroskedasticity and Autocorrelation Consistent Standard Errors (HAC SE) when necessary.[2] In order to compare if there were significant differences between slopes we used a Z-score and its corresponding p-value (equation 2).[3]

$$Z = \frac{\beta_1 - \beta_2}{\sqrt{SE(\beta_1)^2 + SE(\beta_2)^2}} \quad (2)$$

Equation 2. Z-Score for slope comparison

In the primary analysis, Equation 1 was used to estimate the impact of our intervention among all reported poisonings to the Tox Info Suisse, overall and stratified by circumstance (intentional vs accidental).

The estimated regression line for the preintervention period had an intercept determined by $\hat{\beta}_0$ and slope $\hat{\beta}_1$ as shown in Equation 3.

$$\hat{Y}_t\,|_{Post=0} = \hat{\beta}_0 + \hat{\beta}_1 T_t + \hat{\beta}_2 0 + \hat{\beta}_3 T_t 0 = \hat{\beta}_0 + \hat{\beta}_1 T_t \quad (3)$$

Equation 3

© 2020 Martinez-De la Torre A et al. *JAMA Network Open.*

The estimated regression line for the post-intervention period had an intercept determined by $(\hat{\beta}_0 + \hat{\beta}_2)$ while its slope was $(\hat{\beta}_1 + \hat{\beta}_3)$ as shown in Equation 4.

$$\hat{Y}_t\,|_{Post=1} = \hat{\beta}_0 + \hat{\beta}_1 T_t + \hat{\beta}_2 1 + \hat{\beta}_3 T_t 1 \qquad (4)$$
$$= (\hat{\beta}_0 + \hat{\beta}_2) + (\hat{\beta}_1 + \hat{\beta}_3) T_t = \hat{\beta}_{0^*} + \hat{\beta}_{1^*} T_t$$

Equation 4

Additionally, we examined the robustness of our findings by replicating the primary analysis with a quadratic term, as shown in Equation 5.

$$Y_t = \beta_0 + \beta_1 T_t + \beta_2 Post_t + \beta_3 T_t Post_t + \beta_4 T_t^2 Post_t + \varepsilon_t \qquad (5)$$

Equation 5

As a sensitivity analysis, we included ibuprofen as a comparator drug, rerunning the ITS (equation 1) with intervention at Q4 2003 and the changepoint analysis.

References

1. Durbin J, Watson GS. Testing for Serial Correlation in Least Squares Regression. II. *Biometrika*. 1951;38(1/2):159-177. doi:10.2307/2332325

2. White H. A Heteroskedasticity-Consistent Covariance Matrix Estimator and a Direct Test for Heteroskedasticity. *Econometrica*. 1980;48(4):817-838. doi:10.2307/1912934

3. Paternoster R, Brame R, Mazerolle P, Piquero A. Using the Correct Statistical Test for the Equality of Regression Coefficients. *Criminology*. 1998;36(4):859-866. doi:10.1111/j.1745-9125.1998.tb01268.x

© 2020 Martinez-De la Torre A et al. *JAMA Network Open.*

eFigure 1. Interrupted Time Series Analysis for Reported Accidental and Intentional Monointoxication Calls for Acetaminophen in Switzerland Between 2000 and 2018

eFigure 1a. Interrupted time series analysis for reported accidental monointoxication calls for acetaminophen in Switzerland between 2000 and 2018

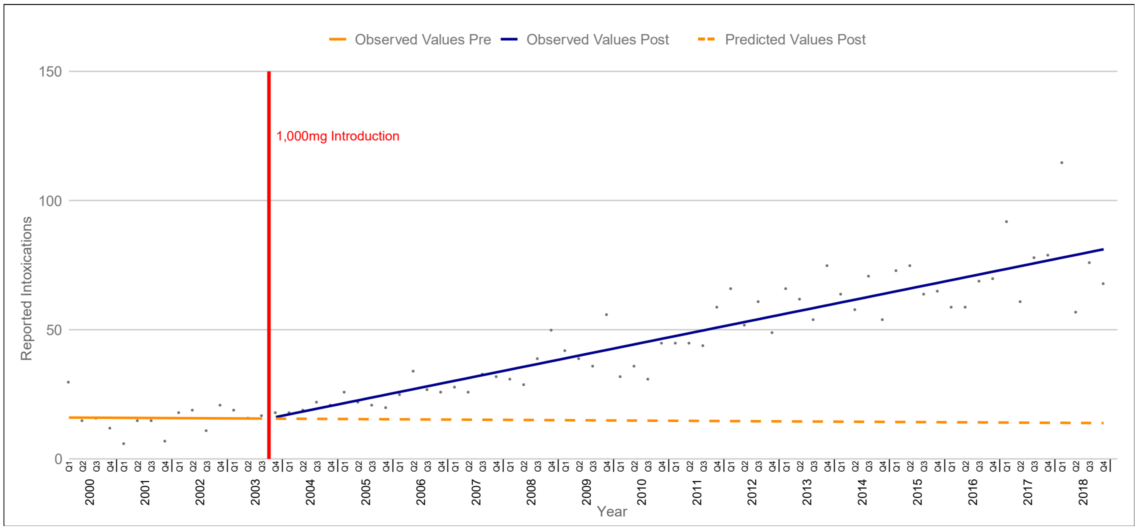

The X axis presents the calendar year and quarter (Q). The Y axis presents the number of reported calls to the National Poison Centre (Tox Info Suisse) for acetaminophen-related poisonings where acetaminophen was the only reported drug (monointoications), and where the circumstance of poisoning was accidental. The red line is the date that the 1,000mg tablets were approved for market sales in Switzerland (October 2003), and identifies the intervention point in the time series analysis. The period to the left (white) is therefore the preintervention period, while that to the right (grey shaded) is the post-intervention period. The solid orange line represents the preintervention trend, the dashed orange line represents the predicted post-intervention period trend from the interrupted time series analysis, and the solid blue line is the observed post-intervention trend.

© 2020 Martinez-De la Torre A et al. *JAMA Network Open.*

eFigure 1b. Interrupted time series analysis for reported intentional monointoxication calls for acetaminophen in Switzerland between 2000 and 2018.

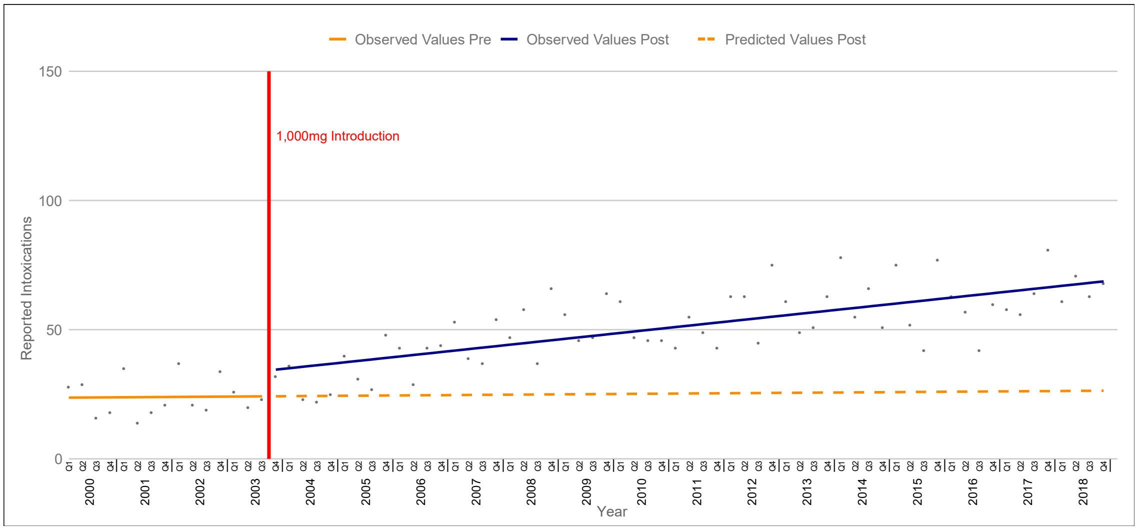

The X axis presents the calendar year and quarter (Q). The Y axis presents the total number of reported calls to the National Poison Centre (Tox Info Suisse) for acetaminophen-related poisonings where acetaminophen was the only reported drug (monointoications), and where the circumstance of poisoning was intentional. The red line is the date that the 1,000mg tablets were approved for market sales in Switzerland (October 2003), and identifies the intervention point in the time series analysis. The period to the left (white) is therefore the preintervention period, while that to the right (grey shaded) is the post-intervention period. The solid orange line represents the preintervention trend, the dashed orange line represents the predicted post-intervention period trend from the interrupted time series analysis, and the solid blue line is the observed post-intervention trend.

© 2020 Martinez-De la Torre A et al. *JAMA Network Open.*

eFigure 2. Interrupted Time Series Analysis With a Quadratic Specification for All Reported Intoxication Calls for Acetaminophen in Switzerland Between 2000 and 2018

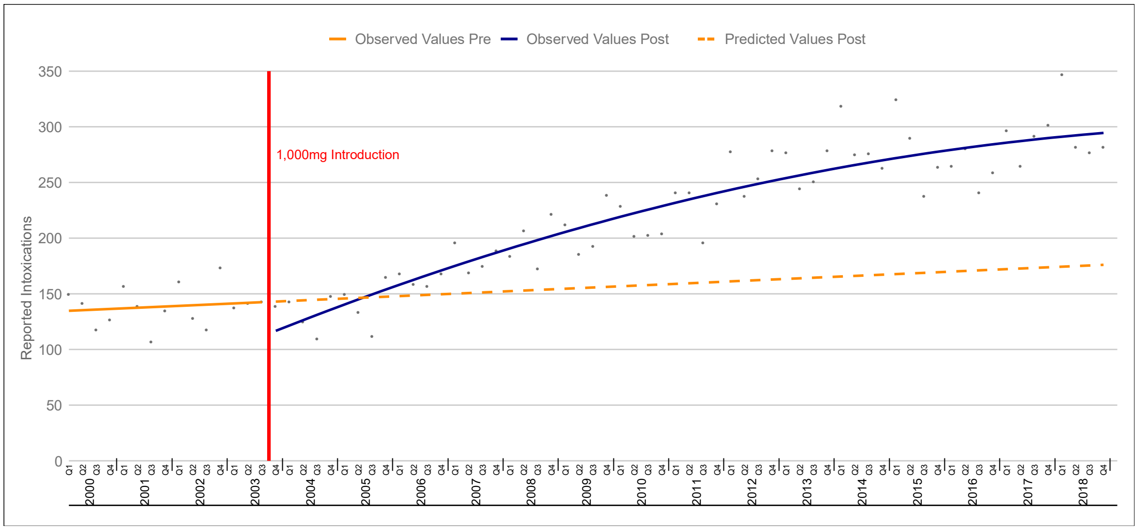

The X axis presents the calendar year and quarter (Q). The Y axis presents the number of reported calls to the National Poison Centre (Tox Info Suisse) for acetaminophen-related poisonings where acetaminophen was the only reported drug (monointoications), and where the circumstance of poisoning was accidental. The red line is the date that the 1,000mg tablets were approved for market sales in Switzerland (October 2003), and identifies the intervention point in the time series analysis. The period to the left (white) is therefore the preintervention period, while that to the right (grey shaded) is the post-intervention period. The solid orange line represents the preintervention trend, the dashed orange line represents the predicted post-intervention period trend from the interrupted time series analysis, and the solid blue line is the observed post-intervention trend with a quadratic fit.

© 2020 Martinez-De la Torre A et al. *JAMA Network Open.*

eFigure 3. Interrupted Time Series Analysis With a Quadratic Specification for Reported Accidental and Intentional Monointoxication Calls for Acetaminophen in Switzerland Between 2000 and 2018

eFigure 3a Interrupted time series analysis with a quadratic specification for reported accidental monointoxication calls for acetaminophen in Switzerland between 2000 and 2018.

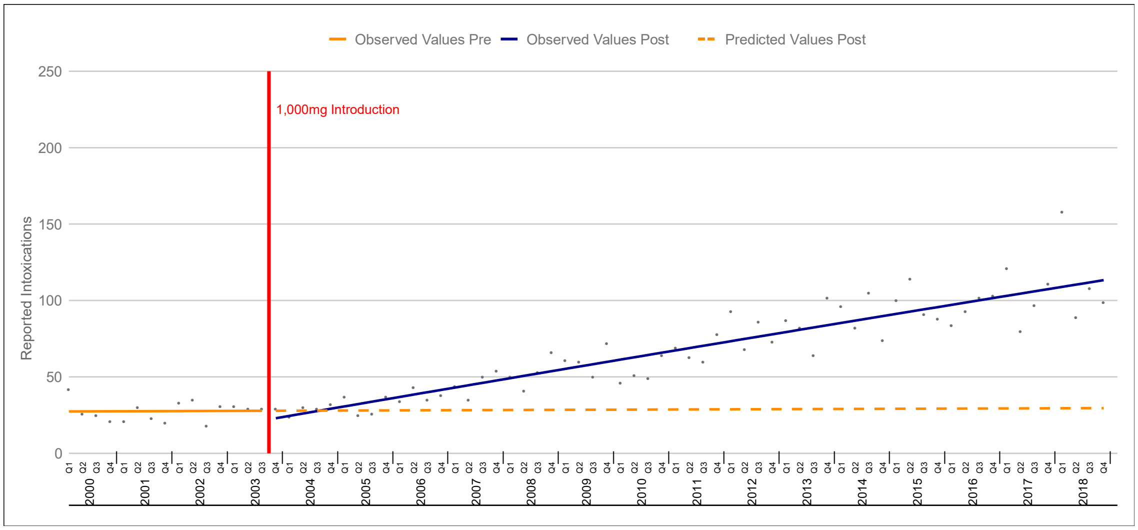

The X axis presents the calendar year and quarter (Q). The Y axis presents the total number of reported calls to the National Poison Centre (Tox Info Suisse) for acetaminophen-related poisonings where acetaminophen was the only reported drug (monointoications), and where the circumstance of poisoning was accidental. The red line is the date that the 1,000mg tablets were approved for market sales in Switzerland (October 2003), and identifies the intervention point in the time series analysis. The period to the left (white) is therefore the preintervention period, while that to the right (grey shaded) is the post-intervention period. The solid orange line represents the preintervention trend, the dashed orange line represents the predicted post-intervention period trend from the interrupted time series analysis, and the solid blue line is the observed post-intervention trend with a quadratic fit.

© 2020 Martinez-De la Torre A et al. *JAMA Network Open.*

eFigure 3b Interrupted time series analysis with a quadratic specification for reported intentional monointoxication calls for acetaminophen in Switzerland between 2000 and 2018.

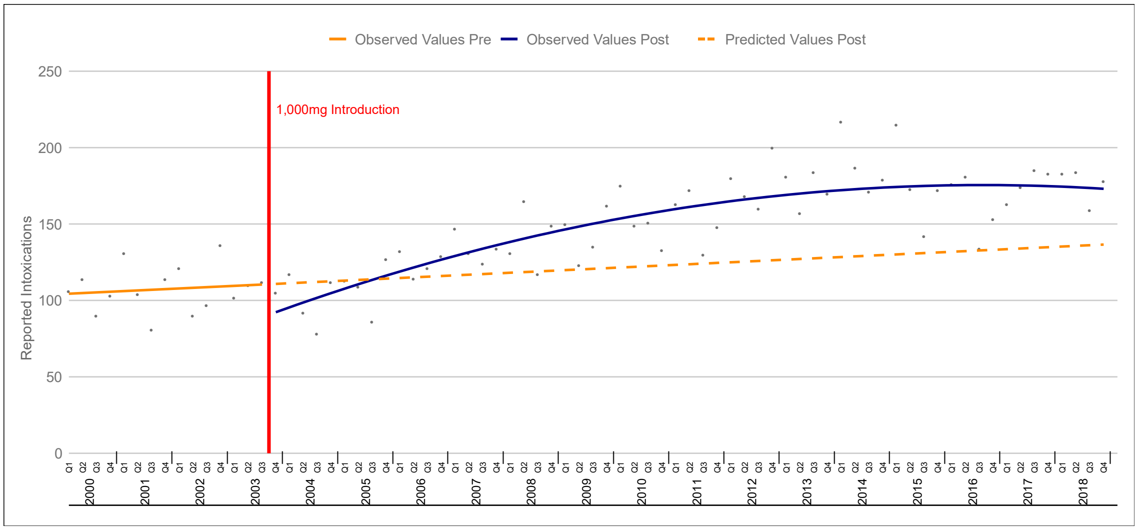

The X axis presents the calendar year and quarter (Q). The Y axis presents the total number of reported calls to the National Poison Centre (Tox Info Suisse) for acetaminophen-related poisonings where acetaminophen was the only reported drug (monointoications), and where the circumstance of poisoning was intentional. The red line is the date that the 1,000mg tablets were approved for market sales in Switzerland (October 2003), and identifies the intervention point in the time series analysis. The period to the left (white) is therefore the preintervention period, while that to the right (grey shaded) is the post-intervention period. The solid orange line represents the preintervention trend, the dashed orange line represents the predicted post-intervention period trend from the interrupted time series analysis, and the solid blue line is the observed post-intervention trend with a quadratic fit.

© 2020 Martinez-De la Torre A et al. *JAMA Network Open.*

eFigure 4. Interrupted Time Series Analysis for All Reported Intoxication Calls for Ibuprofen in Switzerland Between 2000 and 2018

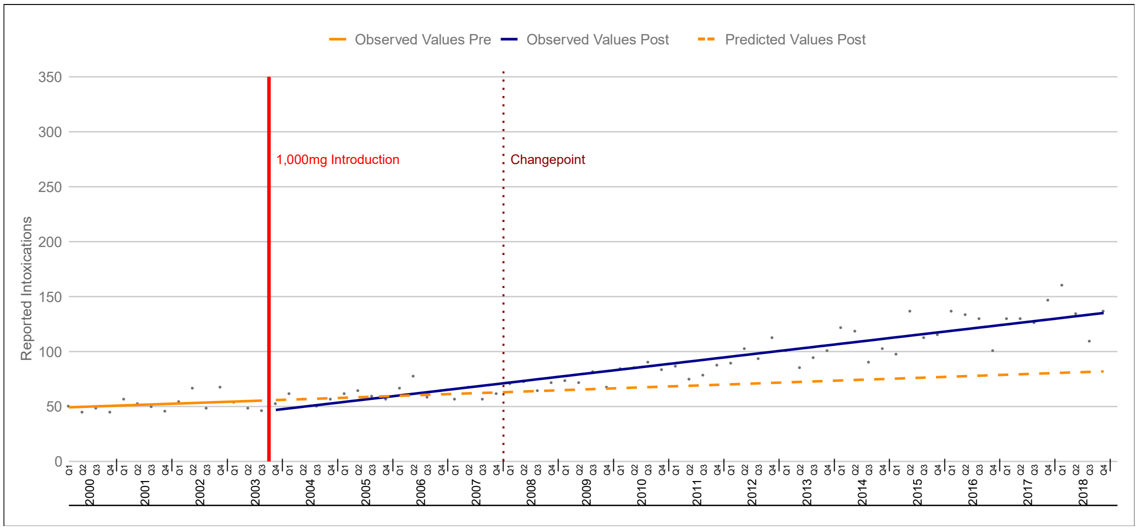

The X axis presents the calendar year and quarter (Q). The Y axis presents the total number of reported calls to the National Poison Centre (Tox Info Suisse) for ibuprofen-related poisonings. The red line is the date that the 1,000mg tablets of acetaminophen were approved for market sales in Switzerland (October 2003), and identifies the intervention point in the time series analysis. The period to the left (white) is therefore the preintervention period, while that to the right (grey shaded) is the post-intervention period. The solid orange line represents the preintervention trend, the dashed orange line represents the predicted post-intervention period trend from the interrupted time series analysis, and the solid blue line is the observed post-intervention trend. The dashed dark-red line indicates where the changepoint analysis identified a statistically significant change in the trend.

© 2020 Martinez-De la Torre A et al. *JAMA Network Open.*

eFigure 5. Total Acetaminophen Tablet Sales in Switzerland, Stratified by Formulation and Year

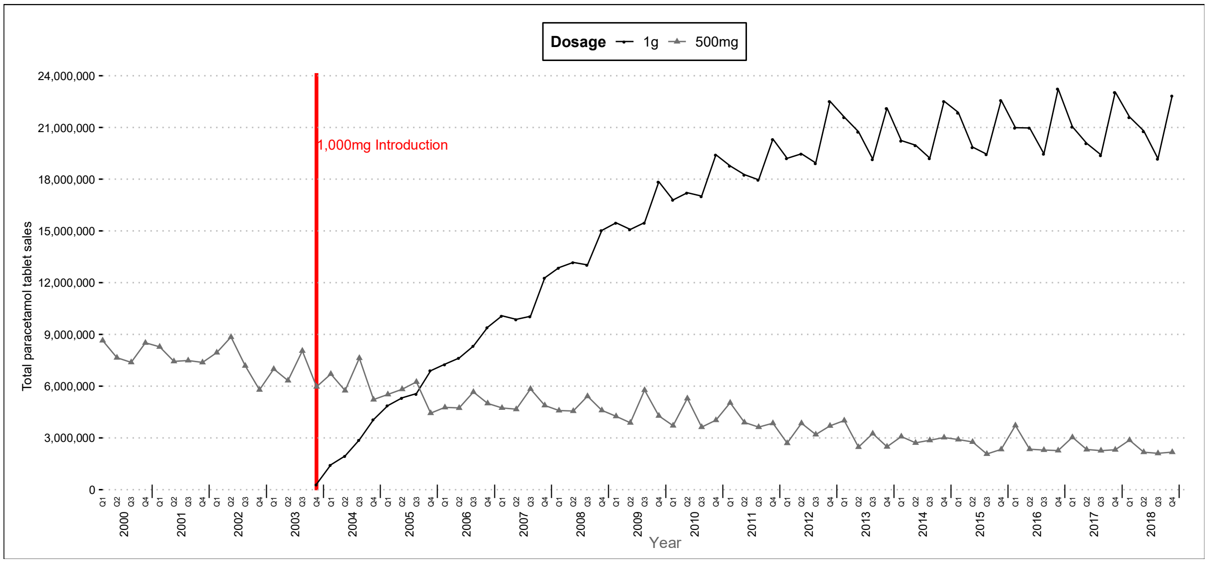

The X axis presents the calendar year and quarter (Q). The Y axis presents the total number of acetaminophen tablets sales. The red line is the date that the 1,000 mg (1 g) tablets were approved for market sales in Switzerland (October 2003). The period to the left (white) is therefore the preintervention period, while that to the right (grey shaded) is the post-intervention period

© 2020 Martinez-De la Torre A et al. *JAMA Network Open.*

eTable 1. Beta Coefficients for Interrupted Time Series Analysis, Stratified by Circumstance and Preintervention and Postintervention
The Z-Score and its corresponding p-value indicate whether there were statistically significant differences between slopes of the Pre and Post period.

			Coefficient	95% CI	p-value	Z-Score	p-value of the Z-score
All calls	Pre	β_0	134.2	117.39, 151.01	<0.001	-3.01	0.002
		β_1	0.55	-1.03, 2.13	0.5		
	Post	β_0^*	88.49	72.43, 104.55	<0.001		
		β_1^*	2.96	2.56, 3.37	<0.001		
All calls – Intentional	Pre	β_0	103.97	91.97, 115.97	<0.001	-1.852	0.064
		β_1	0.43	-0.67, 1.52	0.44		
	Post	β_0^*	89.43	73.65, 105.22	<0.001		
		β_1^*	1.35	0.97, 1.72	<0.001		
All calls – Accidental	Pre	β_0	27.37	20.18, 34.55	<0.001	-3.62	<0.001
		β_1	0.03	-0.76, 0.82	0.94		
	Post	β_0^*	-0.63	-9.1, 7.84	0.88		
		β_1^*	1.5	1.33, 1.68	<0.001		
All Follow-up calls	Pre	β_0	59.38	54.39, 64.38	<0.001	-1.802	0.07
		β_1	0.59	0.12, 1.05	0.014		
	Post	β_0^*	54.13	32.63, 75.63	<0.001		
		β_1^*	0.85	0.33, 1.38	0.001		
Monointoxications - Follow-up calls	Pre	β_0	10.98	6.71, 15.25	<0.001	-1.809	0.07
		β_1	0.06	-0.41, 0.53	0.8		
	Post	β_0^*	9.09	4.41,13.76	<0.001		
		β_1^*	0.46	0.36, 0.55	0.001		

© 2020 Martinez-De la Torre A et al. *JAMA Network Open.*

eTable 2. Demographic Characteristics of 6657 Follow-up Calls Reported to the National Poison Centre in Switzerland for Oral Acetaminophen Tablets Between January 2000 and December 2018, Stratified by Intervention Period

		Overall		Preintervention		Post Intervention	
		Q1 2000 – Q4 2018		Q1 2000 – Q3 2003		Q4 2003 – Q4 2018	
		N	%	N	%	N	%
N		6,657		961		5,696	
Female*		4,680	(70.3)	670	(69.7)	4,010	(70.4)
Unknown*		83	(1.2)	37	(3.9)	46	(0.8)
Mean Age, sd*		28.99	(16.5)	25.12	(13.2)	29.58	(16.9)
Age category*							
	<6	292	(4.4)	41	(4.3)	251	(4.4)
	6-9	14	(0.2)	3	(0.3)	11	(0.2)
	10-15	637	(9.6)	118	(12.3)	519	(9.1)
	16-24	2,337	(35.1)	367	(38.2)	1,970	(34.6)
	25-44	2,103	(31.6)	305	(31.7)	1,798	(31.6)
	45-65	951	(14.3)	76	(7.9)	875	(15.4)
	>65	225	(3.4)	11	(1.1)	214	(3.8)
	unknown	98	(1.5)	40	(4.2)	58	(1.0)
Circumstance of poisoning*							
	Accidental	952	(14.3)	81	(8.4)	871	(15.3)
	Intentional	5,600	(84.1)	866	(90.1)	4,734	(83.1)
	Adverse event	37	(0.6)	7	(0.7)	30	(0.5)
	Unknown	68	(1.0)	7	(0.7)	61	(1.1)
Antidote*							
	Yes	3,581	(53.8)	424	(44.1)	3,157	(55.4)
	No	2,943	(44.2)	522	(54.3)	2,421	(42.5)
	Unknown	133	(2.0)	15	(1.6)	118	(2.1)
Severity							
	Severe*	461	(6.9)	55	(5.7)	406	(7.1)
	Died*	30	(0.5)	1	(0.1)	29	(0.5)
Polyintoxication*		4,649	(69.8)	789	(82.1)	3,860	(67.8)
Monointoxociation*		2,008	(30.2)	172	(17.9)	1,836	(32.2)
Known dose*		4,513	(67.8)	787	(81.9)	3,726	(65.4)
Mean dose in mg, sd*		9,770	(10,390)	7,220	(6,280)	10,310	(10,990)
Median dose in mg, IQR		8,000	(4,000, 12,000)	5,500	(3,000, 10,000)	8,000	(4,000, 13,000)
Dose category±*							
	<4,000 mg	1,307	(19.6)	278	(35.3)	1,029	(27.6)
	4,000 – 9,000 mg	1,946	(29.2)	389	(49.4)	1,557	(41.8)
	≥10,000 mg	1,260	(18.9)	120	(15.3)	1,140	(30.6)

Abbreviations: preintervention: period before 1,000mg acetaminophen introduction, post-intervention: period after acetaminophen introduction, Q: quarter, N: number, sd: standard deviation, mg: milligrams, polyintoxication: more than one substance reported, monointoxication: single substance reported

± proportions calculated among those with a known dose

* p<0.05 between pre- and post-intervention period

© 2020 Martinez-De la Torre A et al. *JAMA Network Open.*

eTable 3. Demographic Characteristics of 193 Children Aged 0 to 9 Years Among Follow-up Monointoxication Calls Reported to the Nation Poison Centre in Switzerland in the Postintervention Period, Stratified by Acetaminophen Formulation

	500 mg Tablets		1000 mg Tablets		Unknown formulation	
	N	%	N	%	N	%
N	31		50		112	
Female	17	(54.8)	29	(58.0)	45	(40.2)
Unknown	2	(6.5)	1	(2.0)	9	(8.0)
Mean Age, sd	2.42	(0.8)	2.44	(1.5)	2.60	(1.5)
Cumulative Dose known	16	(51.6)	26	(52.0)	59	(52.7)
Mean dose in mg, sd	2,140	(1,400)	2,460	(1,500)	1,970	(1,400)
Median dose in mg, IQR	2,250	(900, 3,500)	2,000	(2,000, 3,000	2,000	(1,140, 2,500)
Circumstance						
Accidental	30	(96.8)	50	(100)	108	(96.4)
Intentional	--	--	--	--	3	(2.7)
Unknown	1	(3.2)	--	--	--	--
Antidote						
Yes	8	(25.8)	14	(28)	28	(25.0)
No	23	(74.2)	36	(72)	81	(72.3)
Unknown	--	--	--	--	3	(2.7)
Severe symptoms	--	--	--	--	1	(0.9)

Abbreviations: N: number, sd: standard deviation, mg: milligrams

* p<0.05 between formulations.

© 2020 Martinez-De la Torre A et al. *JAMA Network Open.*

**eTable 4.** Demographic Characteristics of 6851 Monointoxication Calls Reported to the National Poison Centre in Switzerland for Oral Acetaminophen Tablets Between January 2000 and December 2018, Stratified by Intervention Period							
		Overall		Preintervention		Post Intervention	
		Q1 2000 – Q4 2018		Q1 2000 – Q3 2003		Q4 2003 – Q4 2018	
		N	%	N	%	N	%
N		6,851		618		6,233	
Female*		4,400	(64.2)	363	(58.7)	4,037	(64.8)
Unknown*		218	(3.2)	75	(12.1)	143	(2.3)
Mean Age, sd*		20.9	(19.5)	16.0	(14.7)	21.4	(19.8)
Age category*							
	<6	1,643	(24.0)	150	(24.3)	1,493	(24.0)
	6-9	103	(1.5)	12	(1.9)	91	(1.5)
	10-15	716	10.5)	65	(10.5)	651	(10.4)
	16-24	1,489	(21.7)	129	(20.9)	1,360	(21.8)
	25-44	963	(14.1)	63	(10.2)	900	(14.4)
	45-65	471	(6.9)	15	(2.4)	456	(7.3)
	>65	250	(3.6)	7	(1.1)	243	(3.9)
	unknown	1,216	(17.7)	177	(28.6)	1,039	(16.7)
Circumstance of poisoning*							
	Accidental	3,206	(46.8)	237	(38.3)	2,969	(47.6)
	Intentional	3,506	(51.2)	359	(58.1)	3,147	(50.5)
	Adverse event	33	(0.5)	7	(1.1)	26	(0.4)
	Unknown	106	(1.5)	15	(2.4)	91	(1.5)
Known dose*		4,514	(65.9)	428	(69.3)	4,086	(65.6)
Mean dose in mg, sd		9,500	(4,430)	7,300	(7,900)	9,700	(4,650)
Median dose in mg, IQR		6,000	(1,090, 10,000)	5,000	(1,240, 10,000)	6,000	(1,000, 10,000)
Dose category*							
	<4,000 mg	1,911	(27.9)	193	(31.2)	1,718	(27.6)
	4,000 – 9,000 mg	1,507	(22.0)	154	(24.9)	1,353	(21.7)
	10,000 mg	837	(12.2)	63	(10.2)	774	(12.4)
	25,000 mg	259	(3.8)	18	(2.9)	241	(3.9)
	NA	2,337	(34.1)	190	(30.7)	2,147	(34.4)

Abbreviations: preintervention: period before 1000mg acetaminophen introduction, post-intervention: period after acetaminophen introduction, Q: quarter, N: number, sd: standard deviation, mg: milligrams, polyintoxication: more than one substance reported, monointoxication: single substance reported

* $p<0.05$ between pre- and post-intervention period

© 2020 Martinez-De la Torre A et al. *JAMA Network Open.*
